# Application of Ionic Liquids in the Microwave-Assisted Extraction of Proanthocyanidins from *Larix gmelini* Bark

**DOI:** 10.3390/ijms13045163

**Published:** 2012-04-24

**Authors:** Lei Yang, Xiaowei Sun, Fengjian Yang, Chunjian Zhao, Lin Zhang, Yuangang Zu

**Affiliations:** State Engineering Laboratory for Bio-Resource Eco-Utilization, Northeast Forestry University, Harbin 150040, China; E-Mails: vivid66@126.com (X.S.); yangfj@nefu.edu.cn (F.Y.); zcjsj@163.com (C.Z.); zhanglin6600@sina.com (L.Z.)

**Keywords:** ionic liquid, microwave-assisted extraction, proanthocyanidins, *Larix gmelini* bark

## Abstract

Ionic liquid based, microwave-assisted extraction (ILMAE) was successfully applied to the extraction of proanthocyanidins from *Larix gmelini* bark. In this work, in order to evaluate the performance of ionic liquids in the microwave-assisted extraction process, a series of 1-alkyl-3-methylimidazolium ionic liquids with different cations and anions were evaluated for extraction yield, and 1-butyl-3-methylimidazolium bromide was selected as the optimal solvent. In addition, the ILMAE procedure for the proanthocyanidins was optimized and compared with other conventional extraction techniques. Under the optimized conditions, satisfactory extraction yield of the proanthocyanidins was obtained. Relative to other methods, the proposed approach provided higher extraction yield and lower energy consumption. The *Larix gmelini* bark samples before and after extraction were analyzed by Thermal gravimetric analysis, Fourier-transform infrared spectroscopy and characterized by scanning electron microscopy. The results showed that the ILMAE method is a simple and efficient technique for sample preparation.

## 1. Introduction

*Larix gmelini* is a deciduous tree primarily distributed in northeast China, north Sakhalin and east Siberia. It occupies nearly 55% of Great Khingan and Lesser Khingan, China [1]. With physical properties such as rigidness, straight grain and corrosion resistance, *L. gmelini* has been widely used for furniture and building. As a result, large quantities of bark are produced every year as a side product. In recent years, proanthocyanidins have been found in large quantities in *L. gmelini* bark and have been recognized as a multipurpose natural component with great economic potential and environmental value, attracting increasing attention [2–5].

Proanthocyanidins, also known as condensed tannins, are mainly procyanidins with (+)-catechin and (−)-epicatechin as constitutive units. They are naturally occurring polymeric phenolic compounds that are widely found in various fruits, vegetables, seeds, flowers and bark. Importantly, numerous pharmacological effects have been reported for proanthocyanidins, e.g., radical scavenging [6,7], anti-oxidative [8], antimicrobial [9], antiviral [10], anti-inflammatory [11] and anti-carcinogenic effects [12], as well as cardiotonic and anti-arteriosclerotic activities [13,14].

Traditionally, commonly used solvents, including water, methanol, ethanol, acetone, acetic ether and some mixed solvents are used in the extraction of proanthocyanidins from *L. gmelini* bark. And the extraction is usually achieved by using traditional methods such as heating reflux extraction and homogenated extraction [2,4,5]. However, use of these organic solvents is problematic in the extraction of proanthocyanidins due to their toxicity, volatility and flammability. In addition, the traditional extraction involves a complicated working procedure that increases the cost, repeated distillations prolong the heating time and accelerate oxidation of the extract. Thus, the design of safe and environmentally benign extraction solvents and process has an increasingly important role in the development of sample preparation for the analytical determination.

Ionic liquids have been proposed as greener alternatives to traditional organic solvents. Ionic liquids, also known as molten salts, are a new class of non-molecular ionic solvents with a melting point fixed at or below 100 °C. Ionic liquids have some unique chemical and physical properties, for example negligible vapor pressure, thermal and chemical stability, wide liquid range, no inflammability, and no ignition point [15–17], and they are easily recyclable. Ionic liquids could alleviate environmental pollution and improve the selectivity and the extraction efficiencies of compounds in separation technologies [18–20]. Ionic liquids have shown potential as solvents in the extraction of various useful substances from plant samples, such as resveratrol [21,22], piperine [23], aesculin and aesculetin [24], camptothecin and 10-hydroxycamptothecin [25], magnolol and honokiol [26], fangchinoline and tetrandrine [27], carnosic acid and rosmarinic acid [28]. To our best knowledge, the extraction of bioflavonoid complexes proanthocyanidins with an ionic liquid as the solvent has not yet been reported in the literature.

In recent years, microwave-assisted extraction (MAE) has been widely applied in the extraction of active constituents in plants. MAE has many advantages such as convenience, less time consuming, and high efficiency [29–31]. Ionic liquids are very suitable for MAE because the ionic liquids can rapidly and effectively absorb the microwave energy. Adding the ionic liquid into the extraction system can improve the speed of the extraction process. It is therefore of interest to investigate the microwave-assisted extraction of proanthocyanidins using ionic liquids.

In the present study, the potentiality of ionic liquids as alternative solvents in the MAE of proanthocyanidins from *L. gmelini* bark was investigated, and the results compared with conventional extraction methods. Herein, nine ionic liquids with different cations and anions were investigated in an ionic liquid-based microwave-assisted extraction (ILMAE) method. It was found that parameters including the ionic liquid concentration, soak time, solid–liquid ratio, irradiation power and time influenced the extraction yield, and these parameters were optimized systematically. The microstructures and chemical structures of *L. gmelini* bark samples before and after extraction were also investigated by Thermal gravimetric analysis (TG), Fourier-transform infrared spectroscopy (FTIR) and characterized by scanning electron microscopy (SEM).

## 2. Results and Discussion

### 2.1. Screening of the Ionic Liquid-Based Extracting Solvent

The structure of ionic liquids has a significant influence on their physicochemical properties, which might greatly affect the extraction yield of target analytes [19]. To seek the optimal ionic liquids for extraction and evaluate their performance in microwave-assisted extraction process, 1-alkyl-3-methylimidazolium-type ionic liquids with different anions and the alkyl chain length of the cation were researched and recorded in this paper.

#### 2.1.1. Anion Effect

**Figure 1:**
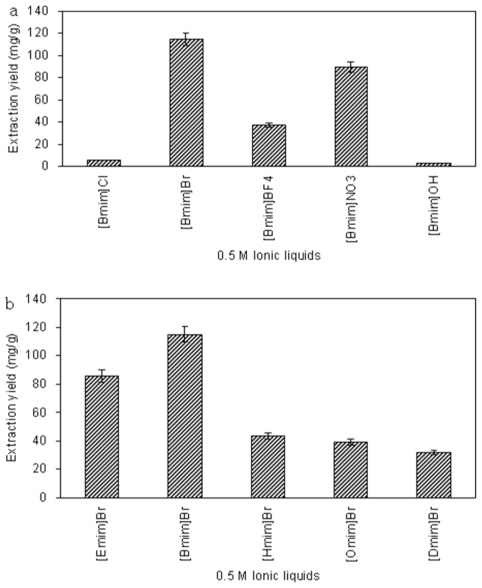
Effect of ionic liquids: anion (**a**) and the alkyl chain length of cation (**b**). Sample: 0.5 g, extractant volume: 10 mL, soak time: 2 h; irradiation power: 230 W; irradiation time: 10 min; ionic liquid concentration: 0.5 M. The extraction yield is expressed as milligram of proanthocyanides extracted per gram bark.

The anion identity is thought to strongly influence an ionic liquid’s properties, particularly affecting water miscibility of ionic liquids [32]. Thus, *n*-methylimidazolium based ionic liquids with five different anions (Cl^−^, Br^−^, BF_4_^−^, NO_3_^−^ and OH^−^) were studied and differences in their extraction yield were readily apparent, as shown in Figure 1a. All of the ionic liquids tested were sufficiently hydrophilic to dissolve in any proportion with water. The results showed that the ionic liquids based on NO_3_^−^ and Br^−^ were the more efficient of the liquids tested for proanthocyanidins, with Br^−^ being the most efficient. This result indicates that extraction yield of proanthocyanidins is anion-dependent, which is similar to observed in previous studies [33,34].

#### 2.1.2. Effect of the Alkyl Chain Length of the Ionic Liquid Cation

In order to gain better extraction yields of the target analytes simultaneously, we subsequently screened the five ionic liquids with increasing alkyl chain length. With the anion Br^−^, a series of 1-alkyl-3-methylimidazolium cations including Emim^+^, Bmim^+^, Hmim^+^, Omim^+^ and Dmim^+^ were evaluated and the results are shown in Figure 1b. The results implied that the extraction yield of proanthocyanidins increased slightly with the increasing alkyl chain length from ethyl to butyl, and the extraction yield decreased rather than increased when the alkyl chain length of the cation was increased from butyl to dodecyl.

Having optimized both the anion and cation of the ionic liquid, [Bmim]Br was selected for subsequent extraction parameter optimization studies.

### 2.2. Optimization of the Extraction Conditions

The univariate method was used to optimize the following parameters: soak time, solid–liquid ratio, irradiation power, irradiation time, ionic liquid concentration and number of extraction cycles.

#### 2.2.1. Effect of Soak Time

**Figure 2:**
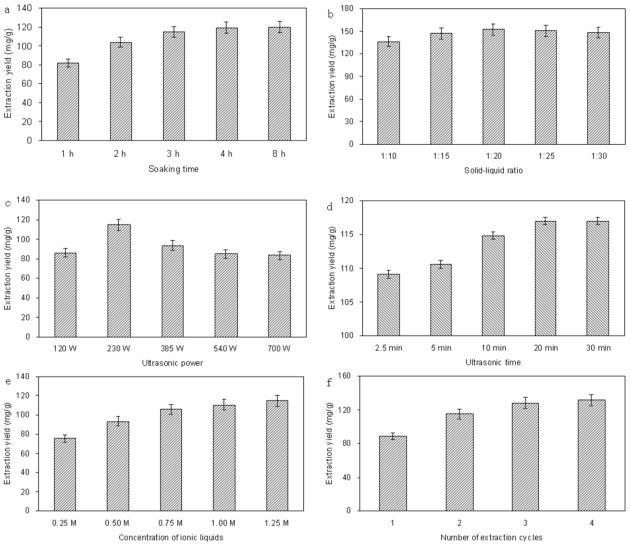
Optimization of extraction conditions: (**a**) 0.5 g of dried sample was mixed with 10 mL 0.5 M [Bmim]Br, and then soaked for different times (1.0, 2.0, 3.0, 4.0 and 8.0 h) before the suspension was extracted for 10 min by MAE (230 W). Extract once; (**b**) 0.5 g of dried sample was mixed with 0.5 M [Bmim]Br with different solid–liquid ratios (1:10, 1:15, 1:20, 1:25 and 1:30 *w*/*v*) and then soaked for 3.0 h before the suspension was extracted for 10 min by MAE (230 W). Extract once; (**c**) 0.5 g of dried sample was mixed with 10 mL 0.5 M [Bmim]Br, and then soaked for 3.0 h, before the suspension was extracted for 10 min by MAE at different ultrasound powers (120, 230, 385, 540 and 700 W). Extract once; (**d**) 0.5 g of dried sample was mixed with 10 mL 0.5 M [Bmim]Br, and then soaked for 3.0 h, before the suspension was extracted for different times (2.5, 5, 10, 20 and 30 min) by MAE (230 W). Extract once; (**e**) 0.5 g of dried sample was mixed with 10 mL [Bmim]Br of different concentration (0.25, 0.50, 0.75, 1.00 and 1.25 M), and then soaked for 3.0 h before the suspension was extracted for 10 min by MAE (230 W). Extract once; (**f**) 0.5 g of dried sample was mixed with 10 mL 1.25 M [Bmim]Br, and then soaked for 3.0 h before the suspension was extracted for 10 min by MAE (230 W), with different number of extraction cycles (1, 2, 3, 4 times). The extraction yield is expressed as milligram of proanthocyanides extracted per gram bark.

Experiments were conducted by soaking the dry bark powder in the ionic liquid solution for 1, 2, 3, 4 or 8 h before MAE. Figure 2a shows the effect of soaking the 0.5 g sample powder in 10 mL 0.5 M [Bmim]Br on the extraction of proanthocyanidins from *L. gmelini* bark at room temperature. We can see the substantial increase in extraction yield obtained after soaking the bark. To extract proanthocyanidins from the cellular structure, the solvent must have access to the cellular compartments where the proanthocyanidins are located. Because the sample is dry powder, sufficient soak time is indispensable to absorb sufficient microwave energy when carrying out the extraction. The extraction yield of proanthocyanidins increased significantly when the soak time was 0–3 h, but longer soak times did not significantly increase the yields of proanthocyanidins. Therefore, in order to save time, the soak time was set to 3 h.

#### 2.2.2. Effect of Solid–Liquid Ratio

The solid–liquid ratio is an important factor and was also studied to optimize extraction yield. Large solvent volumes could make the procedure difficult and lead to unnecessary waste, while small volumes may lead to incomplete extraction. A series of experiments were carried out with different solid–liquid ratios (1:10, 1:15, 1:20, 1:25 and 1:30 g mL^−1^) to evaluate the effect of the solid–liquid ratio. As shown in Figure 2b, the extraction yield increased evidently with the increase of the solvent volume for solid–liquid ratio of up to 1:20. The smaller the ratio of solid-liquid, the more sufficient the contact between sample matrixes and [Bmim]Br aqueous solution was, as a result the more proanthocyanidins were obtained. However, it was not conducive to extract proanthocyanidins when the solid-liquid ratio was too small. When the ratio of solid-liquid was changed from 1:20 to 1:30, the higher solvent volumes did not significantly improve the extraction yield. Therefore, a solid–liquid ratio of 1:20 was determined and then used.

#### 2.2.3. Effect of Irradiation Power

Irradiation power is another important parameter in MAE. To examine the effect of the irradiation power on the extraction yield, experiments were carried out at 120, 230, 385, 540 and 700 W. The MAE time was kept constant throughout this experiment at 10 min. Figure 2c shows the effect of irradiation power on extraction yield. When the irradiation power was increased from 120 to 230 W, the extraction yield of the proanthocyanidins increased. However, when the irradiation power increased above 230 W, the extraction yields decreased instead. Because the ionic liquid has a very high capacity for absorbing microwave energy, too high power may cause the plant to be scorched and decrease the extraction yield, while too low microwave power would lead to a too long MAE time. Thus, the irradiation power of MAE was set at 230 W in the following experiments.

#### 2.2.4. Effect of Irradiation Time

To optimize irradiation time, extractions were carried out at 230 W with various microwave irradiation times up to 30 min and the results are shown in Figure 2d. It is shown that the extraction yield of proanthocyanidins increased dramatically when the microwave time was increased from 2.5 to 10 min. When the variable was changed from 10 to 30 min, slight improvements were observed. The extraction yield was low during the first 5 min of microwave treatment, indicating that more time was needed for the microwaves to disrupt the cell walls and aid the release of the proanthocyanidins into the solvent. Prolonged application of microwaves, of more than 10 min, did not result in any further significant improvement in extraction yield. It was found that more than 98% of the proanthocyanidins content was extracted during the first 10 min of MAE. Therefore, 10 min was set for all subsequent experiments.

#### 2.2.5. Effect of [Bmin]Br Concentration

The extraction procedures were carried out in IL’s solution of different concentrations (from 0.25 to 1.25 M) to determine the optimum [Bmin]Br concentration in aqueous solution for MAE of proanthocyanidins. Based on the results shown in Figure 2e, it can be seen that the extraction yield increased in the [Bmin]Br concentration range of 0.25–1.25 M. This is because with the addition of [Bmim]Br, both the solubility and the extracting capacity of the solvent were enhanced, meanwhile, the capabilities of microwave absorption and microwave conversion were both increased. We propose that the high viscosity of the solvent at high ionic liquid concentrations may lead to poor penetration of the solvent into the plant tissue and high ionic liquids consumption. Finally, 1.25 M [Bmin]Br solution was selected as the optimal ionic liquid concentration.

#### 2.2.6. Effect of Number of Extraction Cycles

The effect of repeated and successive extractions of the residue, *i.e.*, extraction cycle, was studied in this experiment. When continuously extracted 4 times, the total amount was set as 100%. As shown in Figure 2f, with increasing of number of extraction cycles, the extraction yield increased. The extraction yields reached 87.14% ± 3.82% when extracted twice. However, upon further increasing the number of extraction cycles, the extraction yield showed no obvious improvement. To reduce the waste of energy, resource and time, two extractions was chosen for the extraction procedure.

Based on the above experiments, the optimum microwave-assisted conditions were found to be: soak time of 3 h, solid-liquid ratio of 1:20 (*w*/*v*), irradiation power of 230 W, irradiation time of 10 min, [Bmin]Br concentration of 1.25 M and two extraction cycles; 114.86 ± 3.93 mg of proanthocyanidins was extracted per gram of bark used under the optimum conditions.

### 2.3. Comparison of ILMAE Approach with the Reference and Conventional Methods

**Figure 3:**
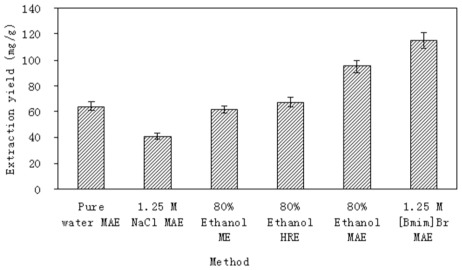
Comparison of ionic liquid based, microwave-assisted extraction (ILMAE) with other extraction methods: 0.5 g of dried sample was mixed with 10 mL pure water, 1.25 M sodium chloride, 80% ethanol or 1.25 M [Bmim]Br. Samples were then soaked for 3.0 h before the extraction. Irradiation time and power: 10 min and 230 W for MAE; extraction time and temperature: 24 h and 25 °C for ME, 4 h and 85 °C for HRE. The extraction yield is expressed as milligram of proanthocyanides extracted per gram bark.

In the present study, MAE, HRE and ME techniques were compared for the yields obtained in the extraction of proanthocyanidins from *L. gmelini* bark. The extraction yield of the proanthocyanidins obtained under different extraction methods using 80% ethanol are summarized in Figure 3. The extraction temperature of HRE was 85 °C, while the extraction temperature used for MAE and ME was room temperature (25 °C), and the extraction times used for MAE, HRE and ME were 10 min, 4 h and 24 h, respectively. The proanthocyanidins extraction yields obtained using MAE methods were higher than those achieved using HRE or ME methods.

In order to further demonstrate the use of ionic liquids, traditional solvents (pure water, 0.5 M NaCl, 80% ethanol) were used to compare with the 1.25 M [Bmim]Br in MAE. All experiments were carried out under the same microwave conditions with the exception of the extractant.

As can be seen in Figure 3, the extraction yield of the proanthocyanidins was 64.08 ± 2.88 mg/g with water, 40.94 ± 4.85 mg/g with 1.25 M NaCl solution and 95.11 ± 4.19 mg/g with 80% ethanol; all lower than the proanthocyanidins extraction yield using 1.25 M [Bmim]Br which was 114.86 ± 3.93 mg/g. The main contributor to proanthocyanidins extraction yield was therefore the ionic liquid rather than water in the ionic liquid–water system. Moreover, salt effects do not play a major role in improving the extraction of proanthocyanidins, because the solvent effect of the ionic liquid was more important in achieving high extraction yields than the salt effect derived from NaCl. This showed that, compared with the traditional solvents, the used ionic liquids could obtain higher extraction yields.

### 2.4. Structural Changes after Extraction

#### 2.4.1. FTIR Analysis

**Figure 4:**
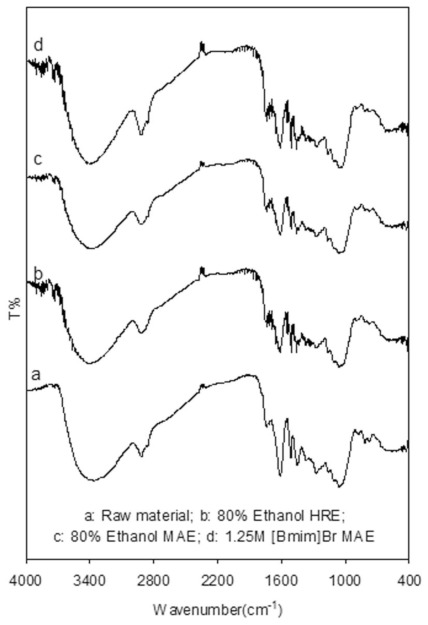
Analysis Fourier-transform infrared spectroscopy (FTIR) spectra of *Larix gmelini* bark samples of raw materials (**a**), 80% ethanol HRE (**b**), 80% ethanol MAE (**c**) and 1.25M [Bmim]Br MAE (**d**).

Since FTIR spectra can provide useful information for identifying the presence of certain function groups or chemical bonds in a molecule or an interaction system, it was applied here to survey the changes in chemical structures of proanthocyanidins before and after extraction by various methods. Analysis FTIR spectra of the unprocessed and processed *L. gmelini* bark sample powders are depicted in Figure 4. The results show that the signal situations and intensity of absorption bands at 3437, 2935, 2864, 1624, 1521, 1296 and 1074 cm^−1^ for *L. gmelini* bark were not apparently changed after 1.25 M [Bmim]Br MAE, compared to those unprocessed, after 80% ethanol HRE and after 80% ethanol MAE methods, which indicated that the chemical structures of carbohydrate compounds, including cellulose, hemicellulose, lignin, and insoluble starch, were unbroken after extraction by the three methods. This result could be related to the fact that water segregated the strong interactions between ionic liquids and carbohydrate compounds of matrixes [35,36]. Hence, the chemical bonding interactions between ionic liquids and sample matrixes were not obvious, which coincides with the study of Liu *et al*. [27].

#### 2.4.2. TG and DTG Analysis

**Figure 5:**
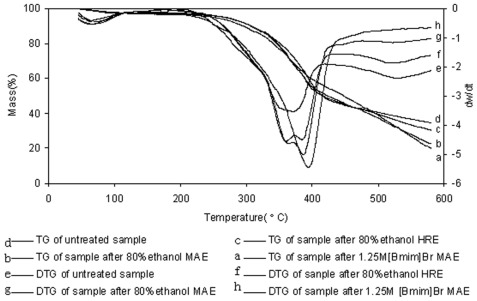
Thermal gravimetric analysis (TG) curves of *Larix gmelini* bark sample powders of raw materials (a), 80% ethanol HRE (b), 80% ethanol MAE (c) and 1.25 M [Bmim]Br MAE (d); TG curves of *Larix gmelini* bark sample powders of raw materials (e), 80% ethanol HRE (f), 80% ethanol MAE (g) and 1.25 M [Bmim]Br MAE (h).

TG and DTG analyses were performed on the samples of unprocessed and processed *L. gmelini* bark powders. As indicated in the TG (a, b, c, d) and DTG (e, f, g, h) curves shown in Figure 5, with a similar trend, as the temperature increased, the weights of all the samples decreased. However, when the temperature was higher than 500 °C, weight loss rates of the four samples were significantly different. The unprocessed sample demonstrated the slowest reduction, while the weight loss was the fastest for the sample processed with 1.25 M [Bmim]Br MAE. This showed the largest gap between the unprocessed sample and 1.25 M [Bmim]Br MAE treated sample, followed by the sample processed with 80% ethanol MAE. According to the DTG curves, double-peak curves at 360–390 °C can be seen in e, f and g, while the 1.25 M [Bmim]Br MAE processed sample (h) only showed a single peak at 390 °C. This indicates that easy to pyrolytic and gasified small molecular components were extracted completely in the sample processed with 1.25 M [Bmim]Br MAE.

#### 2.4.3. Scanning Electronic Microscopy

**Figure 6:**
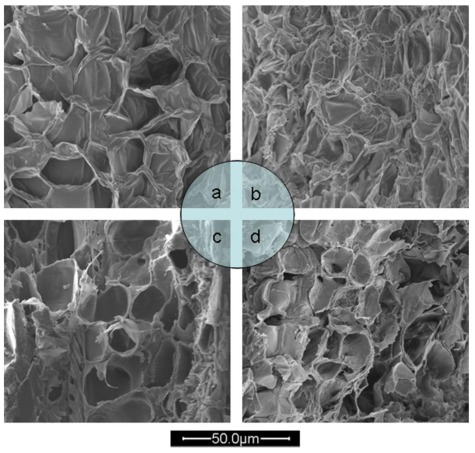
The micrographs of *Larix gmelini* bark samples of raw materials (**a**), 80% ethanol HRE (**b**), 80% ethanol MAE (**c**) and 1.25 M [Bmim]Br MAE (**d**).

The various extraction methods produced distinguishable physical changes of the *L. gmelini* bark samples. Figure 6 shows the micrographs of samples of raw materials, 80% ethanol HRE, 80% ethanol MAE and 1.25 M [Bmim]Br MAE. Before extraction, the presence of numerous full *L. gmelini* bark cells was confirmed (Figure 6a). While after extraction by 80% ethanol HRE, most of them became atrophic and appeared wrinkled, but rupture was rarely observed (Figure 6b). After 80% ethanol MAE, cells and cell walls were greatly affected, as observed by damage of them (Figure 6c). There was more damage seen in Figure 6d, after 1.25 M [Bmim]Br MAE, most cells appeared completely disrupted showing that all the cell walls were finally broken and damaged, resulting into no significant cell shapes. The damage of *L. gmelini* bark sample cells suggests mass transfer of proanthocyanidins into the solvents. The SEM images indicated there were significant differences in the appearance of the *L. gmelini* bark samples cells after different treatment methods.

## 3. Experimental Section

### 3.1. Chemicals and Materials

*L. gemelinii* bark was provided by Mohe Forestry (Heilongjiang, China), and authenticated by Prof. Shao-quan Nie from the State Engineering Laboratory for Bio-Resource Eco-utilization, Northeast Forestry University, China. A voucher specimen was deposited in the herbarium of this Key Laboratory. Its voucher specimen number is 018003001022001. The bark was dried at room temperature for a month and then was powdered into a homogeneous size and sieved (60–80 mesh). The same batch of sample was used here in the experiments.

(+)-Catechin (with purity >98%) standard was purchased from the National Institute for the Control of Pharmaceutical and Biological Products (Beijing, China). All ionic liquids ([Emim]Br, [Bmim]Br, [Hmim]Br, [Omim]Br, [Dmim]Br, [Bmim]Cl, [Bmim]BF_4_, [Bmim]NO_3_ and [Bmim]OH, where Emim = 1-ethyl-3-methylimidazolium, Bmim = 1-butyl-3-methylimidazolium, Hmim = 1-hexyl-3-methylimidazolium, Omim = 1-octyl-3-methylimidazolium), Dmim = 1-decyl-3-methylimidazolium) were obtained from Chengjie Chemical Co. Ltd. (Shanghai, China) and used without further purification. Methanol, hydrochloric acid, vanillin and other reagents are all analytical grade and were obtained from Beijing Chemical Reagents Co. (Beijing, China). Reverse osmosis Milli-Q water (Millipore, Bedford, MA, USA) was used for all solutions and dilutions. All solutions and samples prepared for analysis were filtered through a 0.45 μm nylon membrane (Guangfu Chemical Reagents Co. Tianjin, China).

### 3.2. Microwave-Assisted Extraction Apparatus

A domestic WP700TL 23-K5 microwave-assisted extraction unit (Glanz, Shunde, China) with a 2450 MHz magnetron was used in the extraction step. It was modified in our laboratory with the addition of a water condenser whose wall was coated with PTFE to prevent microwave leakage. The whole system was run at atmospheric pressure and could be employed at the maximum power of 700 W [24].

### 3.3. Ionic Liquids Based Microwave-Assisted Extraction

Ionic liquid-based microwave-assisted extraction was performed in a microwave unit. 0.5 g of dried sample powder was mixed with 10 mL of the various ionic liquid aqueous solutions in a 50 mL flask, and then the suspensions were irradiated under microwave heating. The cation and anion of the ionic liquid, concentration of selected ionic liquid, soak time, solid–liquid ratio, irradiation power and time were systematically optimized in this work to obtain the best extraction efficiency. After each extraction, the obtained extracts were cooled to 25 °C. The extraction yield was expressed as milligram of proanthocyanides extracted per gram bark.

### 3.4. Reference and Conventional Extraction Method

Pure water, 1.25 M sodium chloride and 80% ethanol were selected for use as reference solvents in the MAE of proanthocyanidins from *L. gmelini* bark. The extraction experiments were operated under the optimized conditions except for solvent type. 0.5 g of sample powder was mixed with 10 mL of the above solvents and soaked for 3 h. The suspension was extracted for 10 min by MAE. Irradiation power and the solid–liquid ratio were 230 W and 1:20, respectively. The obtained extracts were cooled to 25 °C and filtered.

80% ethanol was selected as the solvent in conventional maceration extraction (ME) and heat reflux extraction (HRE). The main technical parameters used were the same as above except for extraction time and temperature of 24 h and 25 °C, respectively, for ME, and 4 h and 85 °C, respectively, for HRE.

### 3.5. Vanillin-HCl Method Quantitative Analysis

Proanthocyanidins in extract solution were determined by the standard vanillin-HCl method [37] using (+)-catechin as standard. Briefly, Tto 1.0 mL of the extract solution in a brown tube, 9.0 mL of 2% vanillin/HCl-methanol reagent (2 g vanillin dissolved in 12 N HCl-methanol (1:2) solution to get final volume of 100 mL) was added, immediately capped, mixed for 10 s and incubated at 19–21 °C for 15 min. Absorbance of this solution was measured by spectrophotometer (UV-2550, Shimadzu, Japan) at 500 nm (reference: water) (A_SOLUTION_). Proanthocyanidins content was calculated from the value of (*A*_SOLUTION_) − (*A*_BLANK_) by using a working curve.

The working curve was obtained as follows: 1, 2, 3 mg of (+)-catechin was dissolved in water to a final volume of 10 mL (the standard solution). 1.0 mL of each standard solution was taken in a brown tube and 9.0 mL of 2% vanillin/HCl-methanol reagent was added, immediately capped, mixed for 10 s and incubated at 19–21 °C for 15 min. Absorbance of this solution was measured at 500 nm by spectrophotometer (reference: water) (*A*_CAL_). In case of blank, water was used instead of standard solution (*A*_BLANK_). Working curve was obtained with correcting values: (*A*_CAL_) − (*A*_BLANK_). The working curve was constructed for proanthocyanidins: *Y* = 0.0052 *x* + 0.0164, (*R*^2^ = 0.9974), where *Y* = Absorbance (Abs), *x* = Concentration of reference substance (μg mL^−1^). A good linearity was found for absorbance in the range of 0.107 Abs–1.034 Abs.

### 3.6. Scanning Electron Microscopy

The morphology of the unprocessed and processed *L. gmelini* bark samples were determined by using SEM (FEI, Quanta 200, USA). Particles of representative samples were sputter coated with a thin layer of gold–palladium (5–10 nm; 10 mA; 30 s) at room temperature using a sputter coater before the examination.

### 3.7. Fourier-Transform Infrared Spectroscopy

The unprocessed and processed *L. gmelini* bark sample powders were diluted with KBr mixing powder at 1% and pressed to obtain self-supporting disks, separately. Tablets for FTIR measurements were prepared by pressing the powder mixture at a load of 5 tons for 2 min. The FTIR spectra was obtained by MAGNA-IR560 E.S.P (Nicolet, USA) and recorded across a wave number range of 4000–400 cm^−1^ at a resolution of 4 cm^−1^.

### 3.8. Thermogravimetric Analysis

The thermal gravimetric analysis was performed using a Thermogravimetrical Analyser (TGS-2, PerkinElmer, USA). The unprocessed and processed *L. gmelini* bark sample powders were investigated. Approximately 5 mg of the sample powders were heated at a fixed heating rate of 5 °C/min under a nitrogen purge (50 mL/min), and the percentage weight loss of the samples was monitored from 40 to 600 °C.

### 3.9. Statistical Analysis

The way ANOVA test was used to calculate the significance of the differences of extraction yield for the proanthocyanidins. The results of spectrophotometric analysis were expressed as means of extraction yield ± SD.

## 4. Conclusions

In the present study, we propose a novel extracting method for proanthocyanidins from *L. gmelini* bark based on the use of ionic liquids in MAE followed by Vanillin-HCl method analysis and quantification. The MAE conditions were optimized in detail. Considering the effect of both anion and cation, [Bmim]Br was selected for the subsequent evaluation. The optimum conditions of ILMAE were obtained: soak time 3 h, solid-liquid ratio 1:20 (*w*/*v*), irradiation power 230 W, irradiation time 10 min, [Bmim]Br concentration 1.25 M and two extraction cycles. Under these conditions, satisfactory extraction yield of the proanthocyanidins was obtained and 114.86 ± 3.93 mg of proanthocyanidins was extracted per g of bark used. Relative to other methods, the proposed approach provided higher extraction yield and lower energy consumption. The *Larix gmelini* bark samples before and after extraction were analyzed by TG, FTIR and SEM. The method may also prove useful in the development of energy saving and environmentally friendly extraction methods for proanthocyanidins from other plant materials.
